# Dataset on the formation of Thioredoxin interacting protein (Txnip) containing redox sensitive high molecular weight nucleoprotein complexes

**DOI:** 10.1016/j.dib.2019.104893

**Published:** 2019-11-29

**Authors:** Cristiane Lumi Hirata, Shinji Ito, Hiroshi Masutani

**Affiliations:** a Tenri Health Care University, Tenri, Nara, Japan; b Department of Infection and Prevention, Institute for Frontier and Medical Sciences, Kyoto University, Kyoto, Japan; c Medical Research Center, Graduate School of Medicine, Kyoto University, Kyoto Japan

**Keywords:** Txnip, RNA, lncRNA, High molecular weight complex

## Abstract

This dataset is supplementary to the submitted research by Ref. [1]. RNAs were extracted from high molecular weight complexes, prepared with 100 kDa filtration of HEK293 Tet-on cells stably transfected with either F-HA-Txnip-V5-His or control vector. Cells were stimulated with 1 μg/mL doxycycline for 24 h, followed by overnight stimulation with 100 μM 4-thiouridine (4sU), 20 mM glucose, and 1 μM bortezomib for 14h. The extracted RNAs from Txnip overexpressing cells compared with control cells was analyzed by RNA-seq. Differentially expressed mRNAs, long noncoding RNAs (lncRNA) and transcripts of uncertain coding potential (TUCPs) are shown. Gene ontology and KEGG enrichment of these differential expressed RNAs is presented.

**Table:** Specifications Table

Subject	Biochemistry, Genetics and Molecular Biology
Specific subject area	Cancer Research, Endocrinology, Diabetes, and Molecular Biology
Type of data	Table Graph Figure
How data were acquired	The library preparations were sequenced on an Illumina platform
Data format	Raw Analyzed
Parameters for data collection	HEK293 Tet-on cells (control or Txnip) were grown to 70% confluence and stimulated with 1 μg/mL doxycycline for 24 h, 100 μM 4-thiouridine, 20 mM glucose overnight and 1 μM bortezomib for 14 h. The cells were washed with cold PBS and irradiated with 365 nm UV light (0.15 J/cm^2^) for 2 min.
Description of data collection	Following the UV exposure, less soluble nuclear proteins were extracted by resuspending cell pellets with Triton X-100 buffer after hypotonic and hypertonic buffer treatment. 500–600 μg of samples were incubated with 10 mM MgSO_4_, 10 mM CaCl_2_, and 20% v/v of RQI DNase for 10 min at 37 °C. High molecular protein complexes were prepared using an Amicon 100 kDa filter. RNA was extracted from the solution incubated with 1.2 mg/mL Proteinase K at 55 °C, for 30 min, by the RNeasy Mini kit using RQI DNase for DNase digestion. RNA-seq analyses were performed and analyzed by Novogene.
Data source location	Tenri Health Care University Tenri, Nara Japan
Data accessibility	With the article
Related research article	Cristiane Lumi Hirata^1,2^, Shinji Ito^3^, Hiroshi Masutani^1,2^ Thioredoxin interacting protein (Txnip) forms redox sensitive high molecular weight nucleoprotein complexes Archives of Biochemistry and Biophysics

**Table:** 

**Value of the Data** • This data provides differential expression of RNAs in high molecular weight nuclear extracts comparing Txnip overexpressing cells and control cells. • This data is beneficial for understanding the molecular mechanism of Txnip, a critical regulator in Diabetes. • This data is beneficial for understanding the molecular mechanism of Txnip, an important tumor suppressor. • The data provides insight into the role of nuclear RNA in glucose metabolism and cancer research. • The data may lead to reveal the significance of long noncoding RNAs in cancer and diabetes.

## 1 Data

**Fig. 1:**
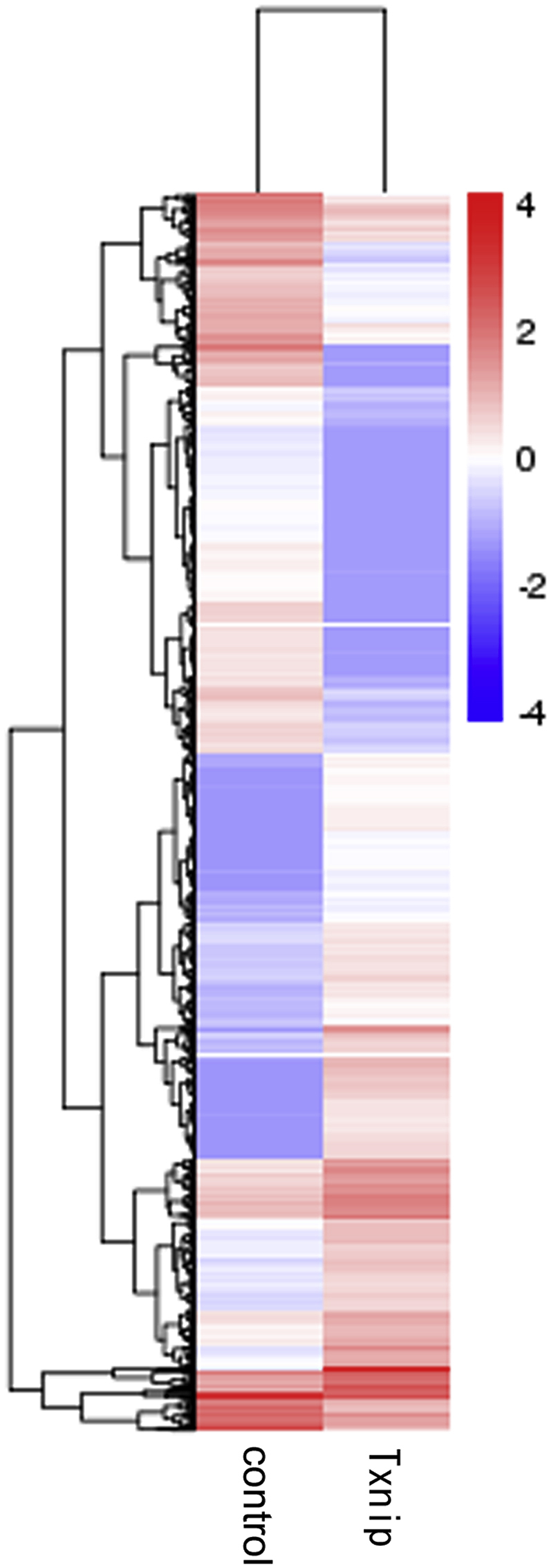
Differentially expressed mRNAs in the complex between Txnip overexpressing cells and control cells. Hierarchical clustering based on Fragments Per Kilobase of transcript sequence per Millions base-pairs sequenced (FPKMs), where log_10_ (FPKM+1) is used for clustering. Red color represents genes with higher expression, while blue represents genes with lower expression.

**Fig. 2:**
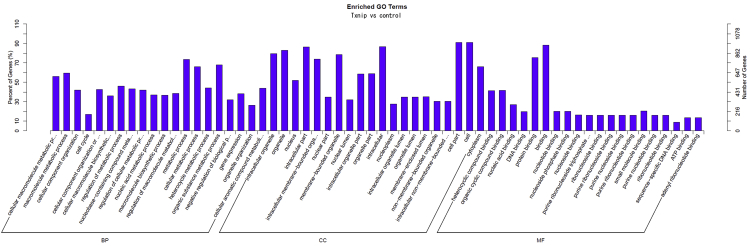
Bar plot of GO enrichment of mRNA target genes comparing Txnip overexpressing and control cells. RNAs from the high molecular complex of Txnip and control cells were analyzed. Horizontal and vertical coordinates represent the enriched GO terms and the number of target genes in that term, respectively. (BP: biological process, MF: molecular function).

Expression of RNAs was analyzed in high molecular weight nuclear complexes from HEK293 Tet-on cells (control or Txnip) [1]. These cells were stimulated with 1 μg/mL doxycycline for 24 h and on the next day, 100 μM 4-thiouridine, 20 mM glucose and 1 μM bortezomib for 14h. Differential expression of mRNA, either up-regulated (Table 1 in supplementary data) or down-regulated (Table 2 in supplementary data) in HEK293 Tet-on cells expressing Txnip compared to control cells is shown. Hierachical clustering of the RNAs is presented in Fig. 1. GO enrichment analyses were performed and are shown in Fig. 2. KEGG enrichment of mRNA target genes comparing Txnip overexpressing and control cells is shown in Table 3 in supplementary data.

**Fig. 3:**
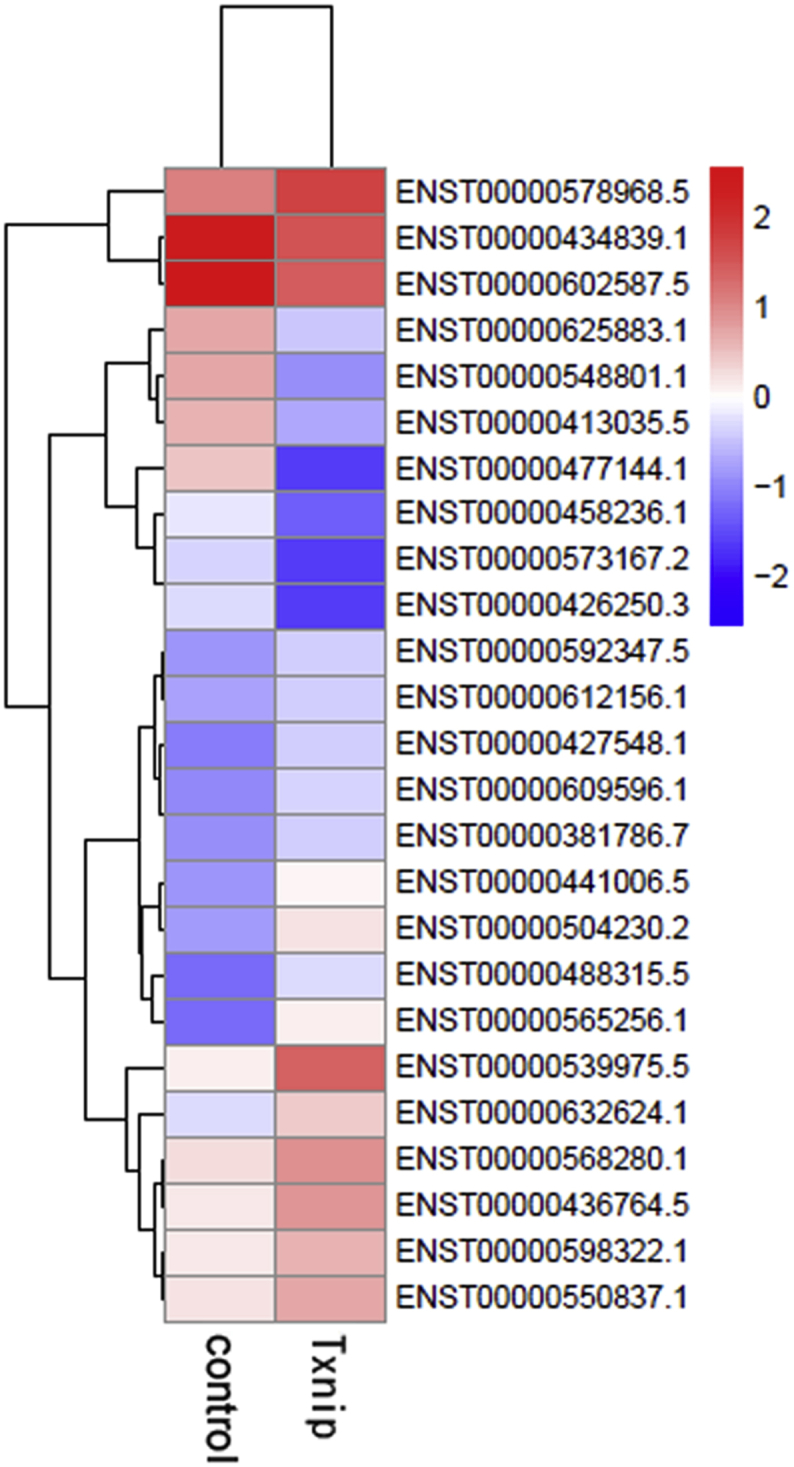
Differentially expressed lncRNAs in the complex between Txnip overexpressing cells and control cells. Hierarchical clustering based on FPKMs, where log_10_ (FPKM+1) is used for clustering. Red color represents genes with higher expression, while blue represents genes with lower expression.

**Fig. 4:**
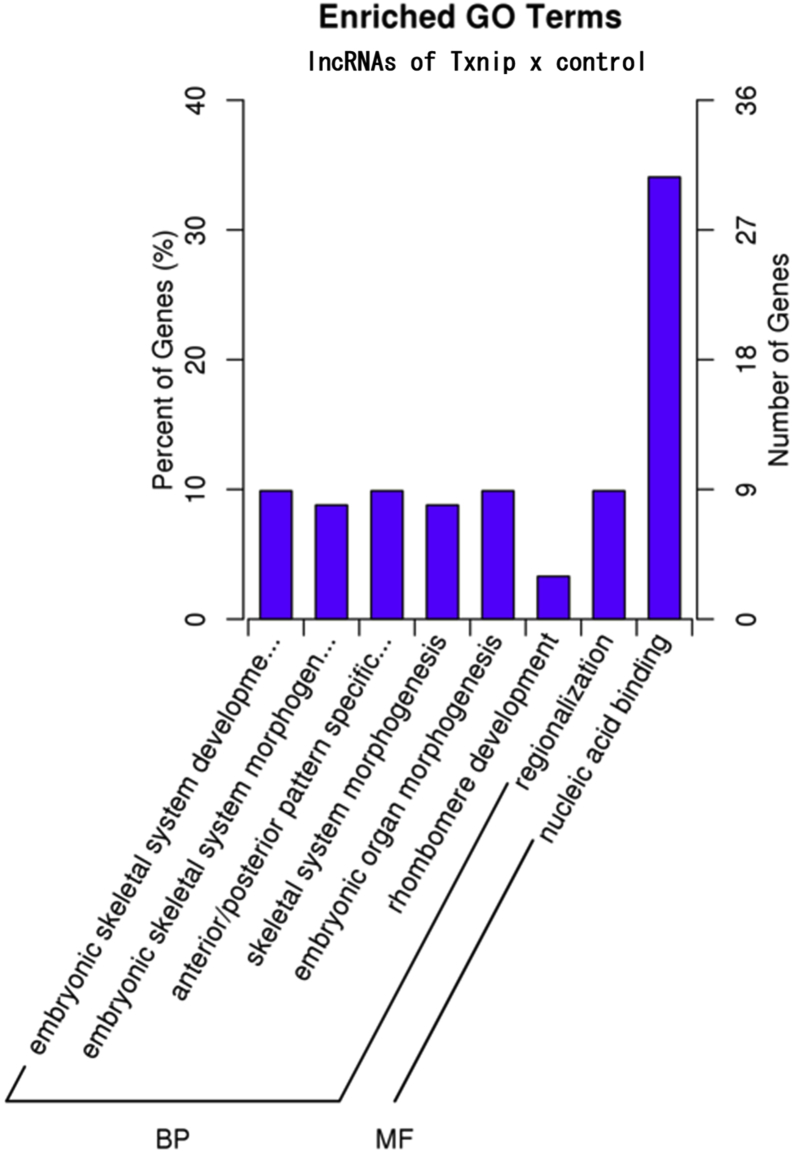
Bar plot of GO enrichment of lncRNA target genes comparing Txnip overexpressing and control cells. RNAs from the high molecular complex of Txnip and control cells were analyzed. Horizontal and vertical coordinates represent the enriched GO terms and the number of target genes in that term, respectively. (BP: biological process, CC: cellular component, MF: molecular function).

We also identified long noncoding RNA (lncRNA), either up-regulated (Table 4 in supplementary data) or down-regulated (Table 5 in supplementary data) in HEK293 Tet-on cells expressing Txnip compared to control cells. Hierachical clustering of the lncRNAs is presented in Fig. 3. GO enrichment analyses were performed and are shown in Fig. 4. KEGG enrichment of lncRNA target genes comparing Txnip overexpressing and control cells is shown in Table 6 in supplementary data.

**Fig. 5:**
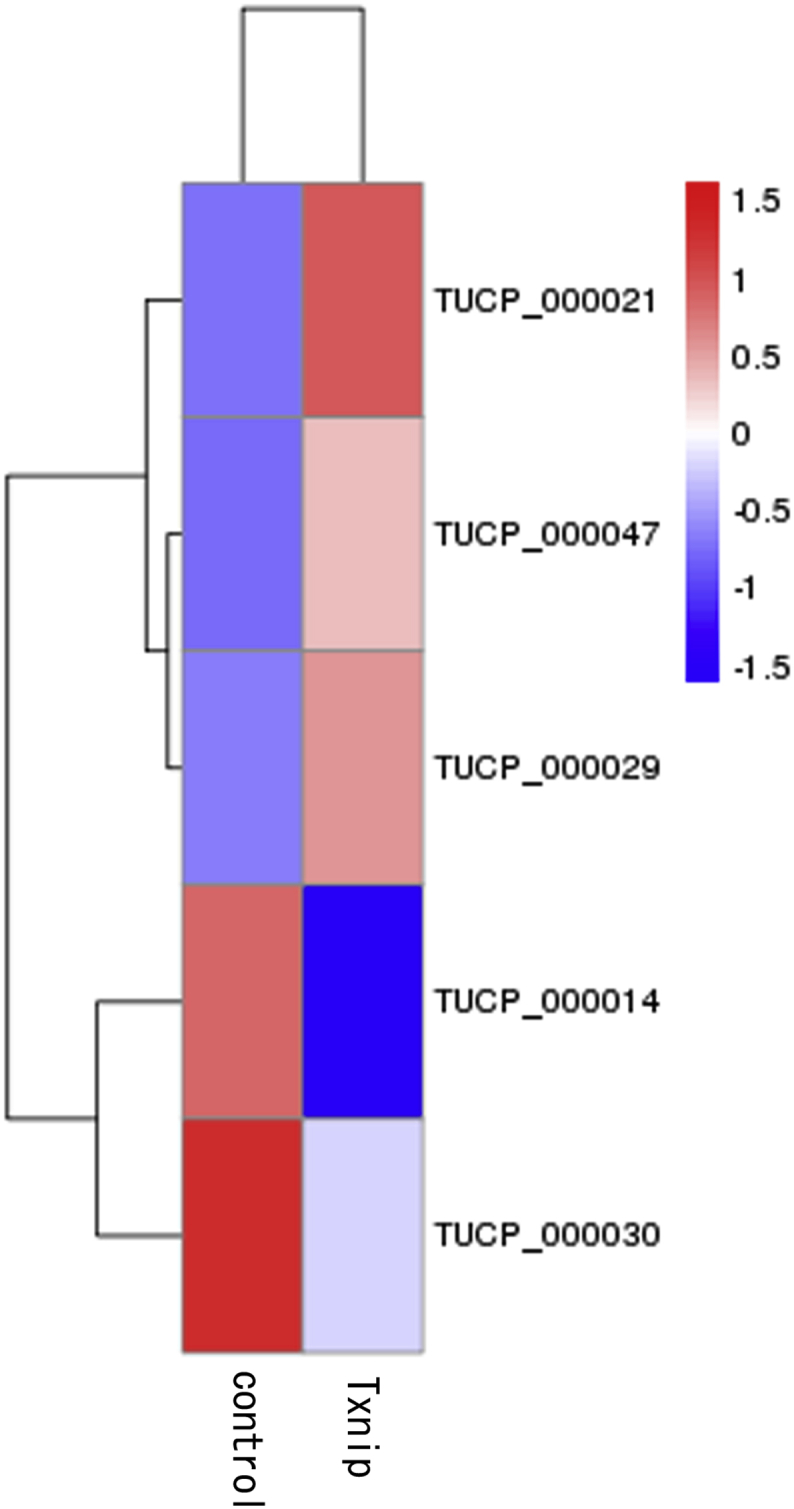
Differentially expressed TUCPs in the complex between Txnip overexpressing and control cells. Hierarchical clustering based on FPKMs, where log_10_(FPKM+1) is used for clustering. Red color represents genes with higher expression, while blue represents genes with lower expression.

Small number of transcripts of uncertain coding potential (TUCPs) were identified. Data presents either up-regulated (Table 7 in supplementary data) RNAs or down-regulated RNAs (Table 8 in supplementary data) in HEK293 Tet-on cells expressing Txnip compared to control cells. Hierachical clustering of the RNAs is presented in Fig. 5.

Alternative Splicing (AS) events comparing Txnip overexpressing and control cells were quantified (Table 9 in supplementary data).

## 2 Experimental design, materials, and methods

### 2.1 Methods for RNA extraction

#### 2.1.1 RNA isolation from protein complexes using 4-thiouridine (4sU) and 365 nm UV light

##### 2.1.1.1 Cell culture, reagents stimulation and UV light exposure

HEK293 Tet-on cells (control or Txnip) were grown in 30 culture plates (10 cm) to 70% confluence and stimulated with 1 μg/mL doxycycline for 24h. On the next day, 100 μM 4-thiouridine (4sU; T384010, Toronto Research Chemicals Inc, Toronto, Canada), 20 mM glucose and 1 μM bortezomib were added to the cells. After 14h, the cells were washed with cold PBS and irradiated with 365nm UV light (0.15 J/cm^2^) for 2 min. Following the UV exposure, cells were scraped and collected in PBS.

##### 2.1.1.2 Cellular fractionation and high molecular weight protein complexes isolation

Less soluble nuclear protein were extracted by resuspending the cell pellet in 3 cell pellet volumes (cpv) of hypotonic buffer (cytosolic fraction), 1.5 cpv of hypertonic buffer (nuclear fraction), and 1 cpv of Triton X-100 buffer (less soluble nuclear protein complexes fraction). After protein quantification, we incubated 500–600 μg of samples with 10 mM MgSO_4_, 10 mM CaCl_2_, and 20% v/v of RQI Dnase for 10 min at 37 °C. To retrieve the high molecular weight protein complexes, we used an Amicon 100 kDa 0.5 mL filter tube kit, and centrifuged tubes at 9000 rpm for 30 min at room temperature (RT). The concentrated high molecular weight protein solution was retrieved by inverting the filter tube into another tube and centrifugation at 2400 rpm for 2 min at RT.

##### 2.1.1.3 Protein digestion, RNA extraction, and RNA-Seq analyses

The above concentrated high molecular protein solution was incubated with 1.2 mg/mL Proteinase K (Qiagen) at 55 °C, for 30 min. We used the RNeasy Mini kit from Qiagen, and adapted the manufacturer protocol for DNase digestion by using RQI DNase instead of DNase I. RNAseq analyses of the RNA samples of HEK293 Tet-on control and HEK293 Tet-on-Txnip cells were performed and analyzed by Novogene.

#### 2.1.2 Methods for RNA-seq

##### 2.1.2.1 RNA quantification and qualification

RNA degradation and contamination was monitored on 1% agarose gels. RNA purity was checked using the NanoPhotometer® spectrophotometer (IMPLEN, CA, USA). RNA integrity was assessed using the RNA Nano 6000 Assay Kit of the Agilent Bioanalyzer 2100 system (Agilent Technologies, CA, USA).

##### 2.1.2.2 Library preparation for lncRNA sequencing

A total amount of 2 μg RNA per sample was used as input material for the RNA sample preparations. Firstly, ribosomal RNA was removed by Epicentre Ribo-zeroTM rRNA Removal Kit (Epicentre, USA), and rRNA free residue was cleaned up by ethanol precipitation. Subsequently, sequencing libraries were generated using the rRNA-depleted RNA by NEBNext® UltraTM Directional RNA Library Prep Kit for Illumina® (NEB, USA) following manufacturer's recommendations. Briefly, fragmentation was carried out using divalent cations under elevated temperature in NEBNext First Strand Synthesis Reaction Buffer (5*X*). First strand cDNA was synthesized using random hexamer primer and M-MuLV Reverse Transcriptase (RNaseH-). Second strand cDNA synthesis was subsequently performed using DNA Polymerase I and RNase H. In the reaction buffer, dNTPs with dTTP were replaced by dUTP. Remaining overhangs were converted into blunt ends via exonuclease/polymerase activities. After adenylation of 3′ ends of DNA fragments, NEBNext Adaptor with hairpin loop structure were ligated to prepare for hybridization. In order to select cDNA fragments of preferentially 250–300 bp in length, the library fragments were purified with AMPure XP system (Beckman Coulter, Beverly, USA). Then 3 μl USER Enzyme (NEB, USA) was used with size-selected, adaptor-ligated cDNA at 37 °C for 15 min followed by 5 min at 95 °C before PCR. Then PCR was performed with Phusion High-Fidelity DNA polymerase, Universal PCR primers and Index.

(X) Primer. At last, products were purified (AMPure XP system) and library quality was assessed on the Agilent Bioanalyzer 2100 system.

##### 2.1.2.3 Clustering and sequencing

The clustering of the index-coded samples was performed on a cBot Cluster Generation System using PE Cluster Kit cBot-HS (Illumina) according to the manufacturer's instructions. After cluster generation, the library preparations were sequenced on an Illumina platform and paired-end reads were generated.

#### 2.1.3 Data analysis

##### 2.1.3.1 Quality control

Raw data (raw reads) of fastq format were firstly processed through in-house perl scripts. In this step, clean data (clean reads) were obtained by removing reads containing adapter, reads on containing ploy- N and low quality reads from raw data. At the same time, Q20, Q30 and GC content of the clean data were calculated. All the downstream analyses were based on the clean data with high quality.

##### 2.1.3.2 Mapping to the reference genome

Reference genome and gene model annotation files were downloaded from genome website (ftp://ftp.ensembl.org/pub/release-82/fasta/homo_sapiens/dna/Homo_sapiens.GRCh38.dna.toplevel.fa.gz) directly. Index of the reference genome was built using Bowtie v2.0.6 and paired-end clean reads were aligned to the reference genome using TopHat v2.0.9.

##### 2.1.3.3 Transcriptome assembly

The mapped reads of each sample were assembled by both Scripture (beta 2) [2] and Cufflinks (v2.1.1) [3] in a reference-based approach. Both methods use spliced reads to determine exons connectivity, but with two different approaches. Scripture uses a statistical segmentation model to distinguish expressed loci from experimental noise and uses spliced reads to assemble expressed segments. It reports all statistically expressed isoforms in a given locus. Cufflinks uses a probabilistic model to simultaneously assemble and quantify the expression level of a minimal set of isoforms that provides a maximum likelihood explanation of the expression data in a given locus. Scripture was run with default parameters, Cufflinks was run with ‘min-frags-per-transfrag = 0’ and ‘–library-type’, other parameters were set as default.

##### 2.1.3.4 Coding potential analysis

Picard - tools v1.41 and samtools v0.1.18 were used to sort, remove duplicated reads and merge the bam alignment results of each sample. GATK3 software was used to perform SNP calling. Raw vcf files were filtered with GATK standard filter method and other parameters (cluster: 3L; WindowSize: 35; QD < 2.0 or FS > 60.0 or MQ < 40.0 or SOR > 4.0 or MQRankSum < −12.5 or ReadPosRankSum Ø −8.0 or DP < 10).

##### 2.1.3.5 CNCI

CNCI (Coding-Non-Coding-Index) (v2) profiles adjoining nucleotide triplets to effectively distinguish protein-coding and non-coding sequences independent of known annotations [4]. We use CNCI with default parameters.

##### 2.1.3.6 CPC

CPC (Coding Potential Calculator) (0.9-r2) mainly through assess the extent and quality of the ORF in a transcript and search the sequences with known protein sequence database to clarify the coding and non-coding transcripts [5]. We used the NCBI eukaryotes' protein database and set the e-value ‘1e-10’ in our analysis.

##### 2.1.3.7 Pfam-scan

We translated each transcript in all three possible frames and used Pfam Scan (v1.3) to identify occurrence of any of the known protein family domains documented in the Pfam database (release 27; used both Pfam A and Pfam B) [6]. Any transcript with a Pfam hit would be excluded in following steps. Pfam searches use default parameters of -E 0.001 –domE 0.001 [7].

##### 2.1.3.8 PhyloCSF

PhyloCSF (phylogenetic codon substitution frequency) (v20121028) examines evolutionary signatures characteristic to alignments of conserved coding regions, such as the high frequencies of synonymous codon substitutions and conservative amino acid substitutions, and the low frequencies of other missense and non-sense substitutions to distinguish protein-coding and non-coding transcripts [8]. We build multi-species genome sequence alignments and run phyloCSF with default parameters. Transcripts predicted with coding potential by either/all of the four tools above were filtered out, and those without coding potential were our candidate set of lncRNAs.

##### 2.1.3.9 Conservative analysis

Phast (v1.3) is a software package contains much of statistical programs, most used in phylogenetic analysis [9], and phastCons is a conservation scoring and identification program of conserved elements. We used phyloFit to compute phylogenetic models for conserved and non-conserved regions among species and then gave the model and HMM transition parameters to phastCons to compute a set of conservation scores of lncRNA and coding genes.

#### 2.1.4 Target gene prediction

##### 2.1.4.1 Cis role of target gene prediction

Cis role is lncRNA acting on neighboring target genes. We searched coding genes 10k/100k upstream and downstream of lncRNA and then analyzed their function.

##### 2.1.4.2 Trans role of target gene prediction

Trans role is lncRNA to identify each other by the expression level. While there were no more than 25 samples, we calculated the expressed correlation between lncRNAs and coding genes with custom scripts; otherwise, we clustered the genes from different samples with WGCNA [10] to search common expression modules and then analyzed their function through functional enrichment analysis.

##### 2.1.4.3 Quantification of gene expression level

Cuffdiff (v2.1.1) was used to calculate FPKMs of both lncRNAs and coding genes in each sample [3]. Gene FPKMs were computed by summing the FPKMs of transcripts in each gene group. FPKM means fragments per kilo-base of exon per million fragments mapped, calculated based on the length of the fragments and reads count mapped to this fragment.

##### 2.1.4.4 Differential expression analysis

Cuffdiff provides statistical routines for determining differential expression in digital transcript or gene expression data using a model based on the negative binomial distribution [3]. For biological replicates, transcripts or genes with an P-adjust <0.05 were assigned as differentially expressed. For non-biological replicates, P-adjust < 0.05 and the absolute value of log 2(Fold change) < 1 were set as the threshold for significantly differential expression.

##### 2.1.4.5 GO and KEGG enrichment analysis

Gene Ontology (GO) enrichment analysis of differentially expressed genes or lncRNA target genes were implemented by the GOseq R package, in which gene length bias was corrected. GO terms with corrected Pvalue less than 0.05 were considered significantly enriched by differential expressed genes. KEGG is a database resource for understanding high-level functions and utilities of the biological system, such as the cell, the organism and the ecosystem, from molecular-level information, especially large-scale molecular datasets generated by genome sequencing and other high-throughput experimental technologies (http://www.genome.jp/kegg/). We used KOBAS software to test the statistical enrichment of differential expression genes or lncRNA target genes in KEGG pathways.

##### 2.1.4.6 Alternative splicing analysis

Alternative splicing events were classified to 12 basic types by the software Asprofile v1.0. The number of AS events in each sample was estimated, separately.

## Appendix A Supplementary data

Table 1 Differential Expression (DE) analysis data showing the up-regulated mRNAs in HEK293 Tet-on cells expressing Txnip compared to control cells. FPKMs: Fragments Per Kilobase of transcript sequence for Millions base-pairs sequenced. q-value: adjusted p-value. Table 1

Table 2 Differential Expression (DE) analysis data showing the down-regulated mRNAs in HEK293 Tet-on cells expressing Txnip compared to control cells. FPKMs: Fragments Per Kilobase of transcript sequence for Millions base-pairs sequenced. q-value: adjusted p-value. Table 2

Table 3 KEGG enrichment of mRNA target genes comparing Txnip overexpressing and control cells. Table 3

Table 4 Differential Expression (DE) analysis data showing the up-regulated lncRNAs in HEK293 Tet-on cells expressing Txnip compared to control cells. Table 4

Table 5 Differential Expression (DE) analysis data showing the down-regulated lncRNAs in HEK293 Tet-on cells expressing Txnip compared to control cells. Table 5

Table 6 KEGG enrichment of lncRNA target genes comparing Txnip overexpressing and control cells. Table 6

Table 7 Differential Expression (DE) analysis data showing the up-regulated TUCPs in HEK293 Tet-on cells expressing Txnip compared to control cells. Table 7

Table 8 Differential Expression (DE) analysis data showing the down-regulated TUCPs in HEK293 Tet-on cells expressing Txnip compared to control cells. Table 8

Table 9 Quantification of Alternative Splicing (AS) events comparing Txnip overexpressing and control cells. Both reads span splicing junctions and reads on target exons are taken into account. Table 9
